# *vanA*-consistent phenotype vancomycin-resistant enterococcal colonization is associated with inferior overall survival in acute myeloid leukemia patients undergoing intensive chemotherapy: a single-center retrospective study

**DOI:** 10.1007/s15010-026-02836-5

**Published:** 2026-05-28

**Authors:** Sebastian Wolf, Nico Zeltner, Nele Henze, Julius C. Enssle, Volkhard A. J. Kempf, Johanna Kessel, Maria J.G.T. Vehreschild, Björn Steffen, Thomas Oellerich, Hubert Serve, Michael Hogardt, Sebastian Scheich

**Affiliations:** 1 Department of Medicine 2, Hematology and Oncology, University Hospital, Goethe-University Frankfurt, Frankfurt am Main, Germany; 2 Institute for Medical Microbiology and Infection Control, University Hospital, Goethe-University Frankfurt, Frankfurt am Main, Germany; 3 Department of Medicine 2, Infectious Diseases, University Hospital, Goethe-University Frankfurt, Frankfurt am Main, Germany; 4 Frankfurt Cancer Institute (FCI), Frankfurt am Main, Germany; 5 German Cancer Consortium (DKTK), Partner Site Frankfurt/Mainz, A Partnership Between DKFZ and University Hospital Frankfurt, Frankfurt am Main, Germany; 6 University Cancer Center (UCT), University Hospital, Goethe-University Frankfurt, Frankfurt am Main, Germany; 7 Department I of Internal Medicine, Faculty of Medicine and University Hospital of Cologne, University of Cologne, Cologne, Germany; 8 German Center for Infection Research (DZIF), Partner Site Bonn-Cologne, Cologne, Germany

**Keywords:** Vancomycin-resistant enterococci, *vanA-consistent* phenotype, *vanB-consistent* phenotype, Acute myeloid leukemia, Teicoplanin resistance, ELN 2022 risk classification, Antimicrobial resistance phenotypes

## Abstract

**Background:**

Vancomycin-resistant enterococci (VRE) colonization is common among patients with acute myeloid leukemia (AML) undergoing intensive chemotherapy. Whether the teicoplanin-defined VRE phenotype, specifically *vanA-*consistent (teicoplanin-resistant) versus *vanB*-consistent (teicoplanin-susceptible), is associated with adverse outcomes is unknown.

**Methods:**

We conducted a retrospective single-center cohort study of 192 VRE-colonized AML patients who received intensive induction chemotherapy between 2015 and 2024. *vanA-*consistent phenotype (*n* = 35, 18.2%) was defined as VRE isolates demonstrating vancomycin (MIC > 4 mg/l) and teicoplanin resistance (MIC > 2 mg/L). *vanB*-consistent phenotype (*n* = 157, 81.8%) was defined as VRE isolates susceptible to teicoplanin. The primary endpoint was overall survival (OS). Cox proportional hazards regression estimated the association between VRE phenotype and OS with adjustment for ELN 2022 genetic risk, sex, ECOG performance status, and allogeneic stem cell transplantation. Healthcare-exposure variables were compared between phenotypes, and the interaction between *vanA*-consistent phenotype and cumulative inpatient days before VRE detection was evaluated.

**Results:**

*vanA*-consistent phenotype patients had significantly inferior OS compared with *vanB*-consistent phenotype patients (3-year OS 46.1% vs. 73.2%; log-rank *p* = 0.004). *vanA-*consistent phenotype was strongly associated with adverse-risk ELN 2022 genetics (54% vs. 24%; OR 3.69, *p* < 0.001). Despite this association, *vanA*-consistent phenotype remained associated with OS after adjustment for ELN 2022 risk, sex, ECOG, and allo-SCT (HR 1.85, 95% CI 1.03–3.34, *p* = 0.041). The *vanA*-associated hazard increased with longer cumulative inpatient exposure before VRE detection. No significant differences were observed for infection-related complications between *vanA-* and *vanB-*consistent phenotypes.

**Conclusions:**

*vanA*-consistent phenotype VRE colonization is an adverse prognostic factor in VRE-colonized AML patients, providing prognostic information beyond ELN 2022 genetic risk classification. To our knowledge, this is the first study to demonstrate differential outcomes by teicoplanin-defined VRE phenotype. Despite enrichment for adverse-risk genetics in *vanA*-consistent phenotype patients, this did not fully account for the survival disadvantage. Prospective multicenter validation is warranted.

**Supplementary Information:**

The online version contains supplementary material available at 10.1007/s15010-026-02836-5.

## Introduction

Acute myeloid leukemia (AML) remains one of the most challenging hematological malignancies, with intensive induction chemotherapy forming the backbone of curative-intent treatment for eligible patients. Treatment-related morbidity and mortality during the aplastic phase remain substantial despite advances in supportive care [1–3]. The European LeukemiaNet (ELN) 2022 genetic risk classification remains the cornerstone of prognostic stratification and treatment decision-making [1]. However, non-genetic factors including patient fitness, comorbidities, and infectious complications also contribute importantly to outcomes [4].

Vancomycin-resistant enterococci (VRE) are among the most frequently encountered multidrug-resistant organisms (MDRO) in patients with hematological malignancies [5]. VRE colonization rates exceed 20% in many hematology units [6, 7], and VRE bloodstream infections are associated with significant attributable mortality [8]. Whether the resistance profile within the colonizing VRE isolate further stratifies risk has not been examined directly. The *vanA* and *vanB* resistance gene clusters confer different antibiotic susceptibility patterns, with teicoplanin resistance being the clinically actionable phenotypic marker that distinguishes *vanA*-consistent from *vanB*-consistent isolates on routine antibiograms.

This phenotypic distinction has been well-characterized at the molecular and epidemiological levels [9]. In particular, Germany has experienced a documented epidemiological shift from *vanA* predominance (approximately 4:1 *vanA*:*vanB* ratio) to *vanB* predominance (approximately 1:4 ratio) over the past 15 years [10–12]. This shift was driven by clonal expansion of ST117 *vanB* lineages, while *vanA* has persisted through distinct sequence types including ST80 [12, 13].

Despite study of VRE as a prognostic factor in hematological malignancies, no prior study has stratified clinical outcomes by *vanA-* versus *vanB-*consistent phenotype. Previous work has examined VRE colonization as a risk factor for mortality and infections but did not further stratify based on resistance profiles [14]. Given the distinct molecular epidemiology, resistance profiles, and lineage characteristics of *vanA* and *vanB* VRE, we hypothesized that *vanA*-consistent phenotype, particularly in a *vanB*-predominant setting, may identify a distinct subpopulation with greater cumulative healthcare exposure and frailty. Such patients may be enriched not only for adverse genetics but also for other host factors that influence survival during intensive chemotherapy.

The aim of this study was to investigate whether *vanA*-consistent phenotype VRE colonization is associated with inferior overall survival in AML patients with documented VRE colonization undergoing intensive induction chemotherapy and whether this association is independent of established prognostic factors, particularly ELN 2022 genetic risk classification.

## Patients and methods

### Study design and population

This was a retrospective single-center cohort study conducted at the Department of Medicine, Hematology/Oncology, Goethe University Frankfurt, Germany. All consecutive adult patients (≥ 18 years) with newly diagnosed AML (excluding acute promyelocytic leukemia, as the ATRA/ATO-based therapy regimen yields a different infection and mortality risk profile) who underwent intensive induction chemotherapy and had documented VRE colonization between January 2015 and October 2024 were included. Patients receiving palliative treatment intent or with prior AML-directed therapy were excluded. Patients who underwent allogeneic stem cell transplantation (allo-SCT) during follow-up were retained in the cohort. Allo-SCT enters the primary Cox model as a time-fixed covariate and is used as a stratum in a sensitivity analysis.

### Definitions

VRE colonization was defined as detection of VRE from surveillance rectal swabs. Resistance phenotypes were determined by VITEK 2 automated susceptibility testing according to EUCAST guidelines under strictly quality-controlled criteria (laboratory accreditation according to ISO 15189:2011 standards; certificate number D–ML–13102–01–00). Teicoplanin-resistant isolates were classified as *vanA*-consistent and teicoplanin-susceptible isolates as *vanB*-consistent. We use the qualifier “consistent” because routine genotypic confirmation is not available and there is a potential for phenotype-genotype discordance. ELN 2022 genetic risk classification was assigned according to the European LeukemiaNet 2022 recommendations based on cytogenetic and molecular findings at diagnosis [1]. AML classification was performed retrospectively in accordance with the International Consensus Classification of myeloid neoplasms and acute leukemias (ICC) 2022 [15].

### Clinical data collection

Demographic, clinical, and laboratory data were extracted from electronic medical records. Variables collected included age at diagnosis, sex, AML type (de novo, secondary, therapy-related), ELN 2022 risk group, ECOG performance status with ≥ 2 dichotomized as “high”, Charlson Comorbidity Index (CCI) with ≥ 3 dichotomized as “high”, white blood cell count at diagnosis, karyotype, and whether allogeneic stem cell transplantation (allo-SCT) was performed during the disease course. Infection-related outcomes included active infection at VRE detection, bacteremia due to multidrug-resistant organisms, *Clostridioides difficile* infection, mucositis, death during aplasia, and ICU admission. Healthcare-exposure variables included MDRO co-colonization (any documented multidrug-resistant organism on surveillance or clinical cultures up to and including the date of first VRE detection, VRE itself was not counted), cumulative inpatient days from AML diagnosis to first VRE detection (summed across admissions), cumulative neutropenia days before VRE detection (sum of episode durations during which the absolute neutrophile count was < 0.5 × 10⁹/L, between AML diagnosis and the day before VRE detection).

### Endpoints

The primary endpoint was overall survival (OS), measured from the date of AML diagnosis to death from any cause or last follow-up. The secondary endpoint was relapse-free survival (RFS), measured from the date of complete remission to relapse or death from any cause. Non-relapse mortality (NRM) was defined as death without prior disease relapse.

### Statistical analysis

Baseline characteristics were compared between *vanA*-consistent and *vanB*-consistent phenotype groups using the Wilcoxon rank-sum test for continuous variables and Fisher’s exact test for categorical variables. Survival outcomes were estimated using the Kaplan–Meier method and compared using the log-rank test.

Univariate and multivariable Cox proportional hazards regression were performed to estimate hazard ratios (HR) with 95% confidence intervals (CI). The primary multivariable model (independence model) included *vanA*-consistent status, ELN 2022 risk group, sex, ECOG performance status (0–1 vs. ≥2), and allo-SCT. We report events per variable (EPV) for all multivariable models. The primary model has EPV = 11.7 (70 events/6 covariates). We additionally fit a Firth-penalized Cox model for the primary specification as a small-sample sensitivity analysis to assess potential bias from sparse strata.

To assess effect modification by prior healthcare exposure, we fitted an interaction model adding cumulative inpatient days before first VRE detection and a *vanA*-consistent phenotype × cumulative inpatient-days interaction term to the primary Cox specification. Interaction significance was assessed by likelihood-ratio test comparing models with and without the interaction term.

Competing risks analysis was performed using cumulative incidence functions estimated by the Aalen–Johansen method, with Gray’s test for group comparisons. Fine–Gray subdistribution hazard models were fitted for NRM with relapse as the competing event.

Prespecified sensitivity analyses included restriction to patients aged < 70 years, restriction to *de novo* AML, exclusion of allo-SCT recipients, and landmark analyses at 3, 6, and 12 months. All analyses were performed using R version 4.3.1 with the survival, survminer, cmprsk, and gtsummary packages. A two-sided *p*-value < 0.05 was considered statistically significant.

### Ethics

The study was approved by the institutional ethics committee of Goethe University Frankfurt (approval number: SHN-08–2017). Informed consent was obtained in accordance with institutional requirements for retrospective analyses.

## Results

### Patient characteristics

A total of 192 VRE-colonized AML patients were included, of whom 35 (18.2%) were classified as *vanA*-consistent and 157 (81.8%) as *vanB*-consistent phenotype. Baseline characteristics are presented in Table 1. The median age at diagnosis was 58 years and did not differ between groups. Sex distribution was similar (46% female in *vanA*-consistent vs. 41% in *vanB*-consistent phenotype).

**Table 1:** Baseline patient characteristics by VRE phenotype

	Overall N = 192	vanB-consistent N = 157	vanA-consistent N = 35	p-value
Age at diagnosis, Median (Q1, Q3)	58 (48, 66)	58 (50, 66)	57 (46, 66)	0.55
Age category, n (%)				0.37
<60	104 (54%)	86 (55%)	18 (51%)	
60-70	60 (31%)	46 (29%)	14 (40%)	
>70	28 (15%)	25 (16%)	3 (8.6%)	
Sex, n (%)				0.70
Male	112 (58%)	93 (59%)	19 (54%)	
Female	80 (42%)	64 (41%)	16 (46%)	
ELN 2022 risk group, n (%)				0.002
Favorable	48 (25%)	44 (28%)	4 (11%)	
Intermediate	87 (45%)	75 (48%)	12 (34%)	
Adverse	57 (30%)	38 (24%)	19 (54%)	
AML type, n (%)				0.20
de novo	146 (76%)	120 (76%)	26 (74%)	
sAML	39 (20%)	33 (21%)	6 (17%)	
tAML	7 (3.6%)	4 (2.5%)	3 (8.6%)	
AML-MR/MRC (ICC 2022), n (%)				0.046
Non-MR	147 (77%)	125 (80%)	22 (63%)	
AML-MR	45 (23%)	32 (20%)	13 (37%)	
Normal karyotype, n (%)	108 (56%)	91 (58%)	17 (49%)	0.35
WBC at diagnosis (log10), Median (Q1, Q3)	1.09 (0.42, 1.83)	1.09 (0.42, 1.83)	1.11 (0.41, 1.86)	0.74
CCI (≥3), n (%)				0.82
1-2	37 (19%)	31 (20%)	6 (17%)	
≥3	155 (81%)	126 (80%)	29 (83%)	
ECOG ≥2, n (%)				0.35
0-1	184 (96%)	149 (95%)	35 (100%)	
≥2	8 (4.2%)	8 (5.1%)	0 (0%)	
Diabetes, n (%)	22 (11%)	17 (11%)	5 (14%)	0.56
Liver disease, n (%)	11 (5.7%)	7 (4.5%)	4 (11%)	0.12
Kidney disease, n (%)	6 (3.1%)	4 (2.5%)	2 (5.7%)	0.30
Heart disease, n (%)	38 (20%)	28 (18%)	10 (29%)	0.16
Allogeneic SCT, n (%)	138 (72%)	114 (73%)	24 (69%)	0.68
MDRO co-colonization (any), n (%)	14 (7.3%)	11 (7.0%)	3 (8.6%)	0.72
Cumulative inpatient days (AML→VRE), Median (Q1, Q3)	27 (17, 48)	27 (16, 47)	30 (21, 55)	0.10
Cumulative neutropenia days (before VRE), Median (Q1, Q3)	19 (10, 34)	20 (10, 34)	19 (11, 36)	0.95

Continuous variables are presented as median (interquartile range) and compared using the Wilcoxon rank-sum test. Categorical variables are presented as n (%) and compared using Fisher’s exact test

*AML* acute myeloid leukemia, *AML-MR/MRC* AML with myelodysplasia-related genomic or cytogenetic changes, *CCI* charlson comorbidity index, *ECOG* eastern cooperative oncology group, *ELN* European leukemiaNet, *ICC* international consensus classification, *ICU* intensive care unit, *MDR* multidrug-resistant, *SCT* stem cell transplantation, *VRE* vancomycin-resistant enterococci

WBC at diagnosis missing for 6 patients; cumulative inpatient days missing for 6 patients; cumulative neutropenia days missing for 6 patients

*vanA*-consistent phenotype patients were enriched for adverse-risk genetics (54% vs. 24%) and AML with myelodysplasia-related genomic or cytogenetic aberrations (AML-MR/MRC per ICC 2022, 37% vs. 20%), with corresponding depletion of favorable-risk (11% vs. 28%) and intermediate-risk (34% vs. 48%) disease (*p* = 0.002). The odds ratio for adverse-risk genetics in *vanA*-consistent patients was 3.69 (95% CI 1.62–8.52, *p* < 0.001). AML type, ECOG performance status, CCI, and allo-SCT rates did not differ significantly between groups. Rates of MDRO co-colonization (8.6% vs. 7.0%, *p* = 0.72), cumulative inpatient days from AML diagnosis to first VRE detection median 30 (IQR 22–55) vs. 27 (IQR 16–47) (*p* = 0.10) and cumulative neutropenia days before VRE detection median 19 (IQR 11–36) vs. 20 (IQR 10–34) (*p* = 0.95) did not differ significantly between groups.

**Fig. 1:**
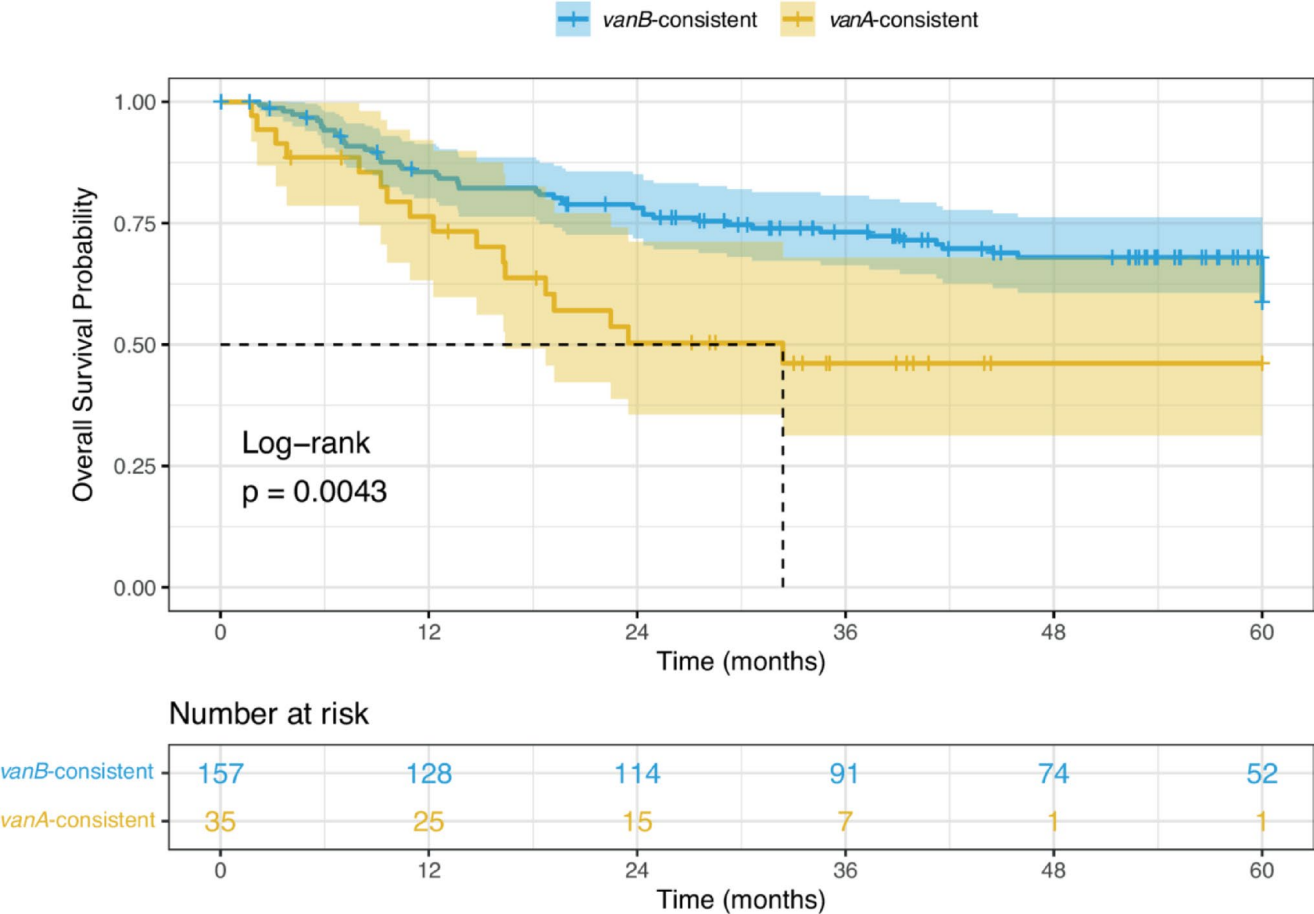
Overall survival by VRE phenotype. Kaplan–Meier curves comparing overall survival between *vanA*-consistent phenotype (*n* = 35) and *vanB*-consistent phenotype (*n* = 157) patients. Shaded areas represent 95% confidence intervals. Dashed lines indicate median survival. The number at risk is displayed below the plot. *p*-value was calculated using the log-rank test

After a median follow-up of approximately five years, 70 deaths (36.5%) were observed, of which 53 were in the *vanB*-consistent group and 17 in the *vanA*-consistent group. Kaplan–Meier analysis showed significantly inferior OS for *vanA*-consistent patients (3-year OS 46.1% vs. 73.2%, log-rank *p* = 0.004; Fig. 1).

In univariate Cox regression, *vanA*-consistent status was associated with a significantly increased hazard of death (HR 2.20, 95% CI 1.26–3.88, *p* = 0.005; Table 2). Other univariate associations included ELN 2022 risk group (intermediate: HR 2.10, *p* = 0.064; adverse: HR 5.52, *p* < 0.001 vs. favorable), secondary AML (HR 2.00, *p* = 0.009), and allo-SCT (HR 0.57, *p* = 0.03).

**Table 2:** Univariate Cox proportional hazards regression for overall survival

Variable	Term	HR	p
van-consistent phenotype	vanA-consistent	2.21 (1.26–3.88)	0.005
Age	60-70	1.10 (0.66–1.85)	0.711
Age	>70	1.18 (0.57–2.44)	0.660
Sex	Female	0.76 (0.47–1.24)	0.273
ELN 2022 risk	Intermediate	2.10 (0.96–4.62)	0.064
ELN 2022 risk	Adverse	5.52 (2.55–11.98)	<0.001
AML type	sAML	2.00 (1.19–3.36)	0.009
AML type	tAML	2.21 (0.79–6.15)	0.129
Normal karyotype	Yes	0.64 (0.40–1.03)	0.067
WBC (log10)		1.07 (0.78–1.46)	0.674
CCI (high)	CCI ≥3	1.45 (0.76–2.76)	0.259
ECOG ≥2	ECOG ≥2	1.26 (0.40–4.03)	0.691
Allogeneic SCT	Yes	0.57 (0.34–0.95)	0.030

Hazard ratios (HR) with 95% confidence intervals (CI) are shown

*CCI* charlson comorbidity index, *ECOG* eastern cooperative oncology group, *ELN* European leukemiaNet, *SCT* stem cell transplantation, *VRE* vancomycin-resistant enterococci, WBC white blood cell count

**Fig. 2:**
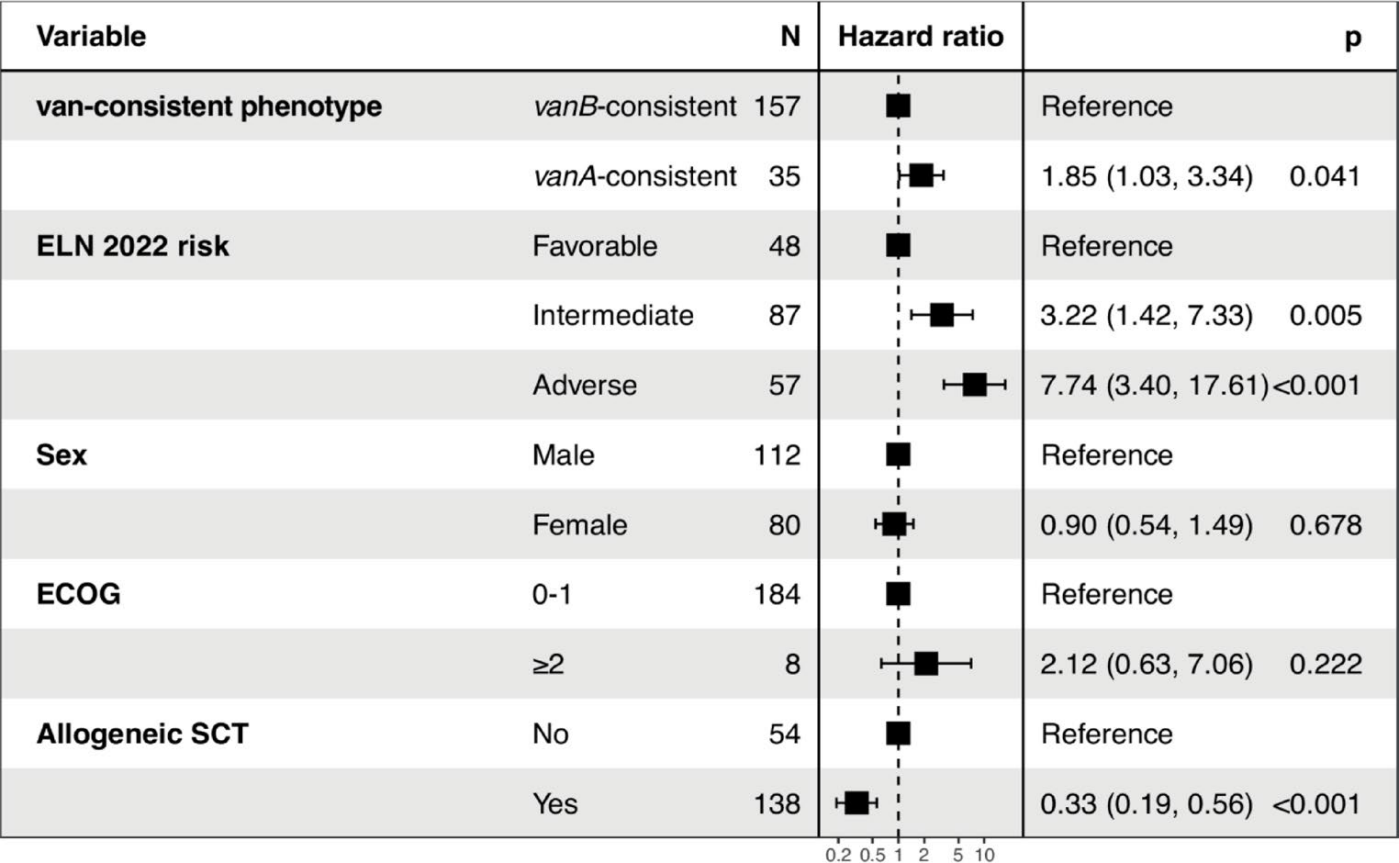
Forest plot of the multivariable Cox regression independence model for overall survival. Hazard ratios with 95% confidence intervals are shown for *vanA*-consistent phenotype status, ELN 2022 risk group, sex, ECOG performance status, and allogeneic stem cell transplantation. The dashed vertical line indicates HR = 1 (no effect)

In the primary multivariable model adjusting for ELN 2022 risk, sex, ECOG status, and allo-SCT, *vanA*-consistent status remained associated with mortality (HR 1.85, 95% CI 1.03–3.34, *p* = 0.041; Fig. 2). The Firth-penalized Cox sensitivity analysis returned a similar estimate (HR 1.88, 95% CI 1.03–3.32, *p* = 0.041). Adding MDRO co-colonization, cumulative inpatient days, cumulative neutropenia days, and ICU admission within 30 days before VRE detection to the primary Cox model left the *vanA*-consistent point estimate stable across models (HR range 1.80–1.88; Table S1). In the most fully adjusted model, the effect size remained similar with reduced precision (HR 1.80, 95% CI 0.98–3.30).

In a sensitivity analysis with allo-SCT stratified, the *vanA*-consistent phenotype effect showed a consistent direction (HR 1.65, 95% CI 0.92–2.98, *p* = 0.094; Table S2).

*vanA*-consistent phenotype and cumulative inpatient days showed a significant interaction in the multivariable model (LRT χ² = 7.40, df = 1, *p* = 0.007). In the interaction model, the *vanA*-consistent hazard ratio rose across the inpatient-day distribution (Fig. S1): 0.74 (0.28–1.94) at the 10th percentile (11 days), 0.92 (0.39–2.16) at the 25th percentile (17 days), 1.32 (0.65–2.68) at the median (27 days), 2.79 (1.45–5.37) at the 75th percentile (48 days), and 6.00 (2.30–15.68) at the 90th percentile (69 days). *vanA*-consistent status was close to neutral in patients with short inpatient exposure at VRE detection and progressively more hazardous in patients with longer exposure.

In exploratory within-ELN 2022 stratum analyses, the estimate was numerically largest in adverse-risk patients (HR 1.86, 95% CI 0.90–3.86, *p* = 0.094), yet numbers were small (Figure S2 and Table S3).

A total of 81 RFS events (42.2%) occurred during follow-up. *vanA*-consistent phenotype patients had numerically worse RFS, but this did not reach statistical significance in univariate analysis (HR 1.55, 95% CI 0.89–2.70, *p* = 0.12; Fig. S3).

**Fig. 3:**
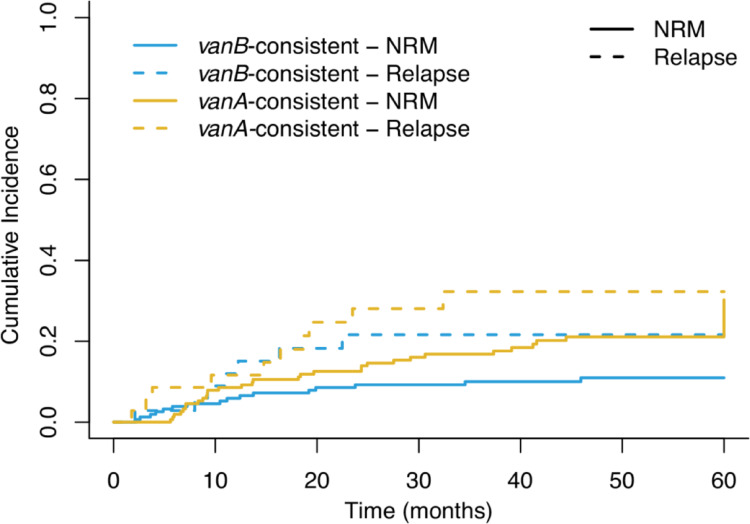
Cumulative incidence of non-relapse mortality (NRM) and relapse by VRE phenotype. Cumulative incidence functions are shown for NRM (solid lines) and relapse (dashed lines) for *vanA*-consistent and *vanB*-consistent phenotype patients in a competing risks framework. *p*-values were calculated using Gray’s test

In competing-risks analysis, the cumulative incidence of NRM was numerically higher in *vanA*-consistent patients compared with *vanB*-consistent phenotype patients (Gray’s test *p* = 0.066; Fig. 3). The cumulative incidence of relapse also was higher in *vanA*-consistent phenotype but did not reach significance (*p* = 0.099).

Rates of active infection at VRE detection were similar between groups (29% vs. 44%, *p* = 0.13) and no significant differences were observed between *vanA*-consistent and *vanB*-consistent phenotype groups for any infection-related outcome (Table 3). Most patients were severely neutropenic (< 0.1 G/L, 57% overall) and the lung was the most common infection site (46%), followed by unknown site (24%), skin/soft tissue (14%), gastrointestinal (8.9%), bloodstream (3.8%), and other (3.8%), with no significant differences between the two groups. *Clostridioides difficile* infection, mucositis, death during aplasia, and ICU admission during or after infection were similar between groups. VRE was identified as the presumed causative pathogen in only 3 of the 79 patients with documented infection.

**Table 3:** Infection-related complications and ICU admissions by VRE phenotype

	vanB-consistent N = 157	vanA-consistent N=35	p-value
Infection at VRE detection, n (%)	69 (44%)	10 (29%)	0.13
Pathogen detection, n (%)	61 (40%)	16 (46%)	0.57
Mucositis, n (%)	7 (4.5%)	3 (8.6%)	0.39
Death in aplasia, n (%)	21 (13%)	4 (11%)	>0.99
ICU ≤30d before VRE detection, n (%)	8 (5.1%)	5 (14%)	0.064
ICU at VRE detection, n (%)	1 (0.6%)	1 (2.9%)	0.33
ICU ≤30d after VRE detection, n (%)	7 (4.5%)	2 (5.7%)	0.67
Neutropenia (<0.5 G/L) at VRE detection, n (%)	117 (75%)	25 (71%)	0.68
Severe neutropenia (<0.1 G/L) at VRE detection, n (%)	92 (59%)	18 (51%)	0.46

Data are presented as n (%) and compared using Fisher’s exact test

*ICU* intensive care unit, *MDR* multidrug-resistant, *VRE* vancomycin-resistant enterococci

Among deceased patients, the cause of death distribution did not differ significantly between groups (*p* = 0.54). AML progression was the most common cause in both groups (*vanA*-consistent phenotype: 53%, *vanB*-consistent phenotype: 66%), followed by sepsis (*vanA*-consistent phenotype: 29%, *vanB*-consistent phenotype: 25%).

Exploratory subgroup analyses showed no clear evidence that the *vanA*-consistent association was restricted to one clinical subgroup. Hazard-ratio estimates were generally directionally consistent across age, ELN 2022 risk, allo-SCT status, comorbidity burden, and sex, although several strata were small (Fig. S4, Table S4).

## Discussion

In this retrospective single-center cohort study of 192 VRE-colonized AML patients, *vanA*-consistent VRE colonization was associated with inferior overall survival, with a hazard ratio of 1.85 after adjusting for ELN 2022 genetic risk, sex, ECOG status, and allo-SCT. This effect was stable across sensitivity models including measured healthcare-exposure. While the role of VRE colonization as a high-risk feature in hematological patients has been consistently reported [14, 16], the differential risk associated with teicoplanin-defined VRE phenotypes has not previously been characterized.

The interaction analysis adds an important qualification. The *vanA*-consistent hazard ratio was close to neutral in patients with short cumulative inpatient exposure at VRE detection and increased with longer exposure, reaching approximately 6 at the 90th percentile of the inpatient-day distribution.

Several explanations remain plausible. Prolonged inpatient stay may maintain antimicrobial selection pressure on the gut microbiome; in *vanA*-consistent carriers, this could increase risk through translocation, downstream infection, or impaired immune recovery. Long inpatient stays may also mark refractory disease, complications, or unmeasured frailty, with *vanA*-consistent colonization acting as a co-marker. A third possibility is survivorship within the early-detected stratum; in short-stay patients, established leukemia risk may dominate outcome, whereas longer-stay patients accumulate non-genetic mortality risks in which the *vanA*-consistent phenotype becomes more informative. Distinguishing between these explanations cannot be resolved with this data set and would require microbiome characterization, detailed antibiotic-exposure data, and external validation.

The timing and setting of this study provide context for interpreting the *vanA*-consistent phenotype effect. Germany experienced a well-documented shift from *vanA* predominance to *vanB* predominance beginning in the early 2010 s [11, 12]. This shift, driven by clonal expansion of ST117/*vanB* lineages, reached its nadir for *vanA* around 2016–2018, with *vanA* comprising only 16.9–22% of VRE isolates in clinical settings [12]. Our study period (2015–2024) captures this expansion of *vanB* dominance.

*vanA*-consistent patients were enriched for adverse-risk ELN 2022 genetics and AML with myelodysplasia-related genomic or cytogenetic aberrations. This pattern suggests that *vanA*-consistent colonization may partly reflect shared antecedents of adverse leukemia biology and prolonged healthcare exposure, rather than a direct effect of the resistance phenotype itself. Patients with secondary, therapy-related, or adverse-risk AML often have longer pre-treatment disease trajectories and more cumulative contact with inpatient care and antimicrobial therapy, which could increase the probability of acquiring a more resistant VRE phenotype. In our cohort, however, the association between *vanA*-consistent phenotype and OS persisted after adjustment for ELN 2022 risk and remained stable after adding measured healthcare-exposure surrogates. The absence of a significant *vanA* × ELN interaction argues against an effect confined to one genetic-risk stratum.

The divergence between OS and RFS is also informative. RFS was numerically inferior in *vanA*-consistent patients but did not reach statistical significance, whereas NRM was numerically higher and infection-specific endpoints did not differ between groups. This pattern is compatible with a non-relapse contribution to the OS signal, but it does not identify the pathway involved. We therefore interpret *vanA*-consistent phenotype as a prognostic marker within VRE-colonized AML patients, potentially integrating leukemia risk, healthcare exposure, and unmeasured host vulnerability.

One biologically plausible explanation involves antibiotic-induced disruption of intestinal colonization resistance. Prior studies have shown that broad antimicrobial exposure can promote intestinal domination by VRE and increase the risk of subsequent VRE bacteremia [17, 18]. Whether this process differs between *vanA*-consistent and *vanB*-consistent colonization is unknown. Differences in the ecological routes by which *vanA*- and *vanB*-associated resistance emerge or persist could contribute to the observed association, but this remains speculative. Our cohort lacks antibiotic-exposure histories and microbiome data; the inpatient-days interaction should therefore be interpreted as hypothesis-generating rather than mechanistic evidence.

This model is consistent with the broader MDRO literature, which demonstrates that colonization with resistant organisms correlates consistently with prolonged hospitalization, prior broad-spectrum antibiotic exposure, vancomycin use, advanced age, and immunosuppression. The intestinal microbiome has been linked to antitumoral immunity and treatment tolerance in hematology patients [19], and disruption may impair immune fitness.

This study has several limitations. As genotyping is not routinely performed for VRE colonization, the *vanA*/*vanB* distinction is based on the teicoplanin resistance phenotype that, although highly correlated, can only serve as a proxy and there is risk for phenotype-genotype discordance. The cohort is restricted to AML patients with documented VRE colonization, and findings apply within this stratum. Conditioning on VRE colonization may induce collider-stratification as colonization itself is a non-random outcome of prior healthcare exposure; thus, extrapolation to the broader AML population is not possible. The temporal and epidemiological context specific to Germany, particularly the *vanA*-to-*vanB* transition, may not generalize to settings where *vanA* remains predominant or where *vanA*/*vanB* proportions differ substantially from those in Germany during this period. Additionally, this is a single-center study, which may be subject to institution-specific practices, screening frequencies, and patient populations that limit generalizability. While we adjusted for major prognostic factors, residual confounding by unmeasured variables, including cumulative antibiotic exposure, specific prior healthcare settings, and microbiome composition cannot be excluded. Detailed antibiotic dosing and duration histories were not consistently retrievable from source records, and inpatient-day-based proxies cannot substitute for measured drug exposure. Finally, with 35 *vanA*-phenotype patients and 17 events, within-stratum and sensitivity analyses had limited statistical power, and we interpreted them as exploratory.

Despite these limitations, the findings have important clinical implications. First, among VRE-colonized AML patients, the vancomycin resistance phenotype, specifically, teicoplanin resistance as a proxy for *vanA* genotype, may augment existing risk stratification. While ELN 2022 genetic classification remains the basis of prognostic assessment, *vanA* phenotype identifies additional high-risk patients, including some with favorable or intermediate genetics, who may warrant closer monitoring and focused anti-infective management.

Second, the strong phenotype-specific association suggests that systematic examination of *vanA*/*vanB* phenotype distribution should be incorporated into future prospective studies of VRE colonization and outcomes across multiple patient populations and geographic regions where phenotype prevalence differs. This would clarify the generalizability of the current findings and whether phenotype-specific risk stratification should inform clinical practice guidelines.

Third, *vanA-*consistent phenotype was associated with OS but not with higher rates of documented acute infection, suggesting that future studies should examine pathways not captured by infection endpoints alone. Microbiome profiling at VRE detection, paired with detailed antibiotic-exposure data, could test whether *vanA-*consistent colonization identifies patients with greater antibiotic-associated microbiome disruption or host vulnerability.

In conclusion, *vanA*-consistent phenotype vancomycin-resistant enterococcal colonization is associated with inferior overall survival in VRE-colonized AML patients undergoing intensive chemotherapy, and the association is not fully accounted for by genetic disease risk, allo-SCT, or measured healthcare-exposure. The effect concentrates in patients with longer cumulative inpatient exposure at VRE detection. The epidemiological context, Germany’s well-documented *vanA*-to-*vanB* shift, provides a setting in which *vanA* colonization in a *vanB*-predominant era identifies a distinct subpopulation. Whether this reflects a biological mechanism, residual confounding by unmeasured illness severity, or survivorship within a heterogeneous group cannot be determined from the present dataset. Prospective multicenter validation across different geographic regions and patient populations is warranted to confirm these findings, clarify generalizability, and assess whether phenotype-stratified risk assessment has clinical value.

## Supplementary Information

Below is the link to the electronic supplementary material.

Supplementary Material 1

Supplementary Material 2

Supplementary Material 3

Supplementary Material 4

Supplementary Material 5

Supplementary Material 6

Supplementary Material 7

Supplementary Material 8

## Data Availability

The datasets generated and analyzed during the current study are available from the corresponding author on reasonable request.

## References

[1] Döhner H, Wei AH, Appelbaum FR, Craddock C, DiNardo CD, Dombret H, et al. Diagnosis and Management of AML in Adults: 2022 ELN Recommendations from an International Expert Panel. Blood [Internet] Blood. 2022. 10.1182/BLOOD.2022016867. [cited 2022 Aug 11]. 35797463 10.1182/blood.2022016867

[2] Taplitz RA, Kennedy EB, Bow EJ, Crews J, Gleason C, Hawley DK, et al. Outpatient management of fever and neutropenia in adults treated for malignancy: American Society of Clinical Oncology and Infectious Diseases Society of America clinical practice guideline update. J Clin Oncol. 2018;36:1443–53. 10.1200/JCO.2017.77.6211. 29461916 10.1200/JCO.2017.77.6211

[3] Taplitz RA, Kennedy EB, Bow EJ, Crews J, Gleason C, Hawley DK, et al. Antimicrobial prophylaxis for adult patients with cancer-related immunosuppression: ASCO and IDSA clinical practice guideline update. J Clin Oncol. 2018;36:3043–54. 10.1200/JCO.18.00374. 30179565 10.1200/JCO.18.00374

[4] Buckley SA, Othus M, Estey EH, Walter RB. The treatment-related mortality score is associated with non-fatal adverse events following intensive AML induction chemotherapy. Blood Cancer J. 2015;5:e276–e276. 10.1038/bcj.2014.97. 25635529 10.1038/bcj.2014.97 PMC5404219

[5] Tatarelli P, Mikulska M. Multidrug-resistant bacteria in hematology patients: emerging threats. Future Microbiol. 2016;11:767–80. 10.2217/fmb-2015-0014. 27196948 10.2217/fmb-2015-0014

[6] Tavadze M, Rybicki L, Mossad S, Avery R, Yurch M, Pohlman B, et al. Risk factors for vancomycin-resistant enterococcus bacteremia and its influence on survival after allogeneic hematopoietic cell transplantation. Bone Marrow Transplant. 2014;49:1310–6. 10.1038/bmt.2014.150. 25111516 10.1038/bmt.2014.150

[7] Liss BJ, Vehreschild JJ, Cornely OA, Hallek M, Fätkenheuer G, Wisplinghoff H, et al. Intestinal colonisation and blood stream infections due to vancomycin-resistant enterococci (VRE) and extended-spectrum beta-lactamase-producing Enterobacteriaceae (ESBLE) in patients with haematological and oncological malignancies. Infection. 2012;40:613–9. 10.1007/s15010-012-0269-y. 22665143 10.1007/s15010-012-0269-y

[8] Weber S, Hogardt M, Reinheimer C, Wichelhaus TA, Kempf VA, Kessel J, et al. Bloodstream infections with vancomycin-resistant enterococci are associated with a decreased survival in patients with hematological diseases. Ann Hematol. 2019;98:763–73. 30666433 10.1007/s00277-019-03607-z

[9] Gorrie C, Higgs C, Carter G, Stinear TP, Howden B. Genomics of vancomycin-resistant *Enterococcus faecium*. Microb Genom. 2019;5:e000283. 10.1099/mgen.0.000283. 31329096 10.1099/mgen.0.000283 PMC6700659

[10] Falgenhauer L, Preuser I, Imirzalioglu C, Falgenhauer J, Fritzenwanker M, Mack D, et al. Changing epidemiology of vancomycin-resistant *Enterococcus faecium*: results of a genome-based study at a regional neurological acute hospital with intensive care and early rehabilitation treatment. Infection Prevention in Practice. 2021;3:100138. 10.1016/j.infpip.2021.100138. 34368749 10.1016/j.infpip.2021.100138 PMC8335922

[11] Liese J, Schüle L, Oberhettinger P, Tschörner L, Nguyen T, Dörfel D, et al. Expansion of Vancomycin-resistant *Enterococcus faecium* in an academic tertiary hospital in Southwest Germany: a large-scale whole-genome-based outbreak investigation. Antimicrob Agents Chemother. 2019;63:e01978-18. 10.1128/AAC.01978-18. 30782988 10.1128/AAC.01978-18 PMC6496047

[12] Correa-Martínez CL, Jurke A, Schmitz J, Schaumburg F, Kampmeier S, Mellmann A. Molecular epidemiology of Vancomycin-Resistant Enterococci bloodstream infections in Germany: a population-based prospective longitudinal study. Microorganisms. 2022. 10.3390/microorganisms10010130. 35056579 10.3390/microorganisms10010130 PMC8777844

[13] Bender JK, Kalmbach A, Fleige C, Klare I, Fuchs S, Werner G. Population structure and acquisition of the vanB resistance determinant in German clinical isolates of *Enterococcus faecium* ST192. Sci Rep. 2016;6:21847. 10.1038/srep21847. 26902259 10.1038/srep21847 PMC4763178

[14] Scheich S, Weber S, König R, Wilke AC, Lindner S, Reinheimer C, et al. Timepoints of vancomycin-resistant Enterococcus colonization predict outcomes of acute myeloid leukemia patients undergoing allogeneic hematopoietic cell transplantation. European Journal of Haematology. 2018;101:620–9. 10.1111/ejh.13151. 10.1111/ejh.13151 30048011

[15] Arber DA, Orazi A, Hasserjian RP, Borowitz MJ, Calvo KR, Kvasnicka H-M, et al. International Consensus Classification of Myeloid Neoplasms and Acute Leukemias: integrating morphologic, clinical, and genomic data. Blood. 2022;140:1200–28. 10.1182/blood.2022015850. 35767897 10.1182/blood.2022015850 PMC9479031

[16] Xie O, Slavin MA, Teh BW, Bajel A, Douglas AP, Worth LJ. Epidemiology, treatment and outcomes of bloodstream infection due to vancomycin-resistant enterococci in cancer patients in a vanB endemic setting. BMC Infect Dis. 2020;20:228. 10.1186/s12879-020-04952-5. 32188401 10.1186/s12879-020-04952-5 PMC7079500

[17] Ubeda C, Taur Y, Jenq RR, Equinda MJ, Son T, Samstein M, et al. Vancomycin-resistant Enterococcus domination of intestinal microbiota is enabled by antibiotic treatment in mice and precedes bloodstream invasion in humans. J Clin Invest. 2010;120:4332–41. 10.1172/JCI43918. 21099116 10.1172/JCI43918 PMC2993598

[18] Taur Y, Xavier JB, Lipuma L, Ubeda C, Goldberg J, Gobourne A, et al. Intestinal domination and the risk of bacteremia in patients undergoing allogeneic hematopoietic stem cell transplantation. Clin Infect Dis. 2012;55:905–14. 10.1093/cid/cis580. 22718773 10.1093/cid/cis580 PMC3657523

[19] Peled JU, Devlin SM, Staffas A, Lumish M, Khanin R, Littmann ER, et al. Intestinal microbiota and relapse after hematopoietic-cell transplantation. J Clin Oncol. 2017;35:1650–9. 10.1200/JCO.2016.70.3348. 28296584 10.1200/JCO.2016.70.3348 PMC5455763

